# Supplementary material for: Impact of the COVID-19 Pandemic on the Therapeutic Continuity among Outpatients with Chronic Cardiovascular Therapies
Source: Int J Environ Res Public Health. 2022 Sep 24;19(19):12101. doi: 10.3390/ijerph191912101 (PMC9566639; doi:10.3390/ijerph191912101)
Supplement: Supplementary file 1 [file ijerph-19-12101-s001.zip › ijerph-1908049-supplementary.pdf]

Impact of the COVID-19 Pandemic on the Therapeutic Continuity among Outpatients with Chronic Cardiovascular Therapies

Manuela Casula [1,2], Federica Galimberti [2], Marica Iommi [3], Elena Olmastroni [1,*], Simona Rosa [4], Mattia Altini [5], Alberico L. Catapano [1,2], Elena Tragni [1] and Elisabetta Poluzzi [3]

[1] Epidemiology and Preventive Pharmacology Service (SEFAP), Department of Pharmacological and Biomolecular Sciences, University of Milan, Via Balzaretti 9, 20133 Milan, Italy
[2] IRCCS MultiMedica, Via Milanese 300, Sesto S. Giovanni, 20099 Milan, Italy
[3] Department of Medical and Surgical Sciences-Pharmacology Unit, University of Bologna, Via Irnerio 48, 40126 Bologna, Italy
[4] Department of Biomedical and Neuromotor Sciences-Hygiene and Biostatistics Unit, University of Bologna, Via San Giacomo, 40126 Bologna, Italy
[5] Romagna Local Health Authority, Emilia-Romagna Region, Via A. De Gasperi 8, 48121 Ravenna, Italy
* Correspondence: elena.olmastroni@unimi.it

Table S1. List of ATC codes for each cohort.

Chronic Treatment	ATC Codes
**Lipid-lowering drugs**	**Class 1 (statins)**: C10AA, C10BA02, C10BA05, C10BA06<br>**Class 2 (PCSK9 inhibitors)**: C10AX13, C10AX14<br>**Class 3 (ezetimibe)**: C10AX09, C10BA02, C10BA05, C10BA06
**Antidiabetics**	**Class 1 (biguanides)**: A10BA, A10BD02, A10BD05, A10BD07, A10BD08, A10BD10, A10BD11, A10BD13, A10BD15, A10BD16, A10BD20, A10BD23<br>**Class 2 (sulfonylureas)**: A10BB, A10BD02, A10BD06<br>**Class 3 (sulfonamides)**: A10BC<br>**Class 4 (alpha glucosidase inhibitors)**: A10BF<br>**Class 5 (thiazolidinediones)**: A10BG, A10BD05, A10BD06, A10BD09<br>**Class 6 (dipeptidyl peptidase 4 (DPP-4) inhibitors)**: A10BH, A10BD07, A10BD08, A10BD09, A10BD10, A10BD11, A10BD13, A10BD19, A10BD21<br>**Class 7 (glucagon-like peptide-1 (GLP-1) analogues)**: A10BJ<br>**Class 8 (sodium-glucose co-transporter 2 (SGLT2) inhibitors)**: A10BK, A10BD15, A10BD16, A10BD19, A10BD20, A10BD21, A10BD23<br>**Class 9 (other blood glucose lowering drugs, excl. insulins)**: A10BX
**Antihypertensives**	**Class 1 (antiadrenergic agents)**: C02<br>**Class 2 (diuretics)**: C03, C07BB, C07CB, C09BA, C09BX01, C09DA, C09XA52<br>**Class 3 (beta blocking agents)**: C07, C07BB, C07CB, C09BX02<br>**Class 4 (calcium channel blockers)**: C08, C09BB, C09BX01, C09DB<br>**Class 5 (agents acting on the renin-angiotensin system)**: C09, C09BA, C09BB, C09BX01, C09BX02, C09DA, C09DB, C09XA52

Figure S1. Example of PDC calculation for an individual with two concurrent therapeutic classes within the same chronic therapy.

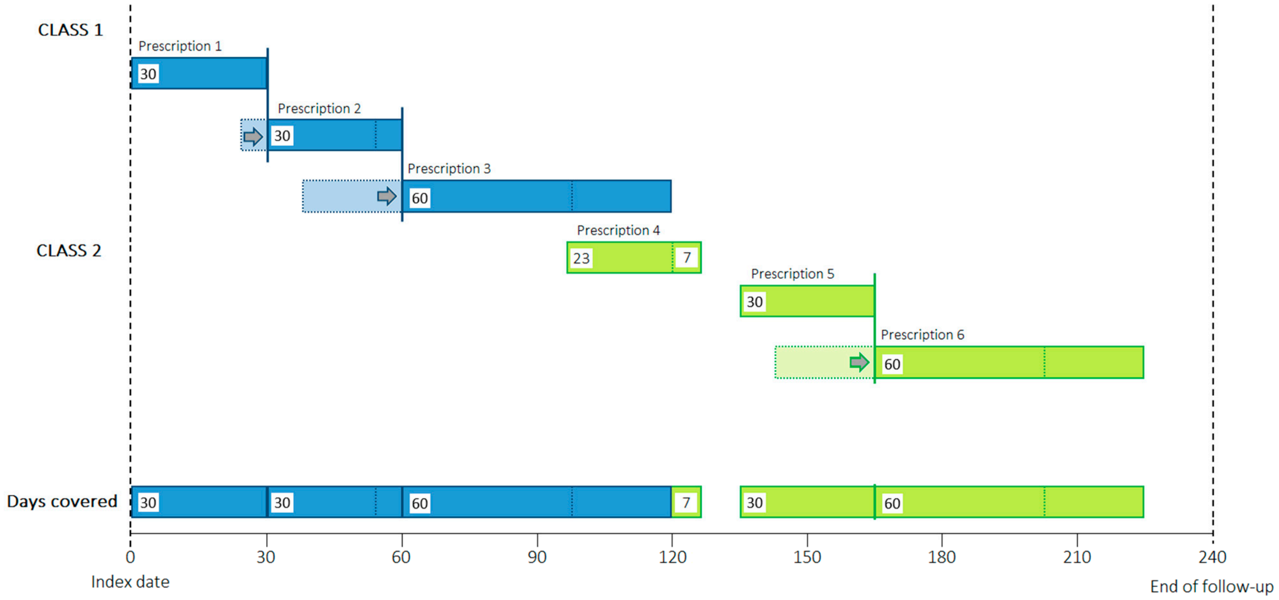

Days of follow-up: 240

Days covered: 30 + 30 + 60 + 7 + 30 + 60 = 217

Proportion of days covered (PDC): $\frac{217}{240} = 0.90$

Figure S2. Timeline of the study.

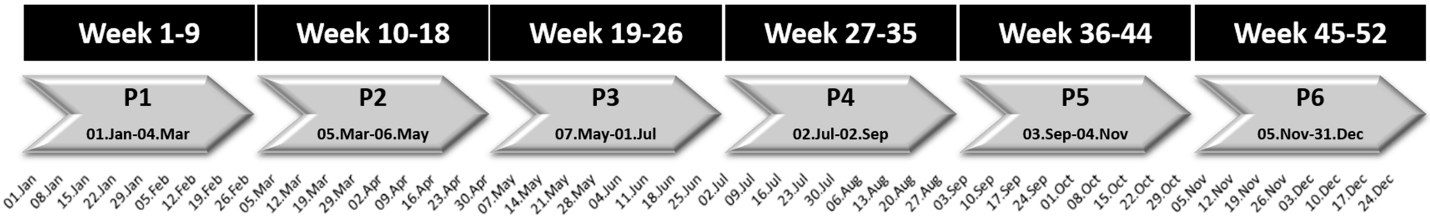

Figure S3. Difference in dispensed packages per 1000 patients between study and control periods by sex.

(A) Lipid-lowering drugs

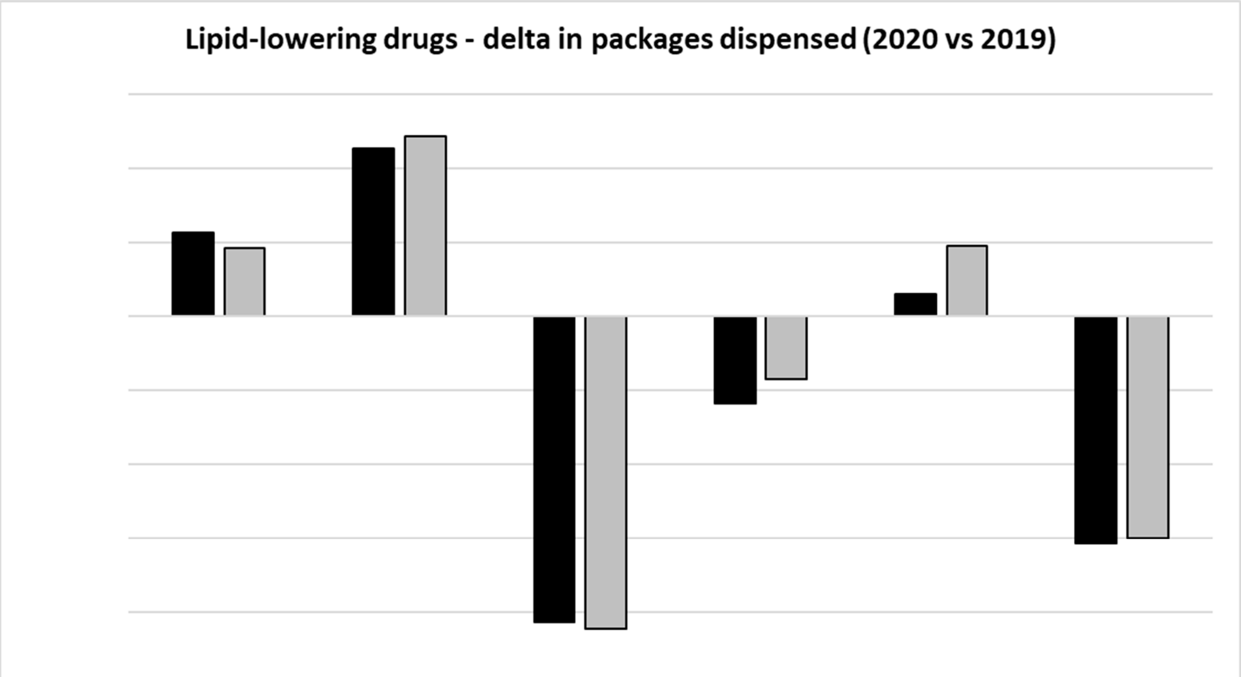

	ΔP1	ΔP2	ΔP3	ΔP4	ΔP5	ΔP6
■ Male	2.28%	4.54%	-8.28%	-2.37%	0.59%	-6.16%
□ Female	1.87%	4.87%	-8.45%	-1.70%	1.93%	-5.99%

(B) Antidiabetic drugs

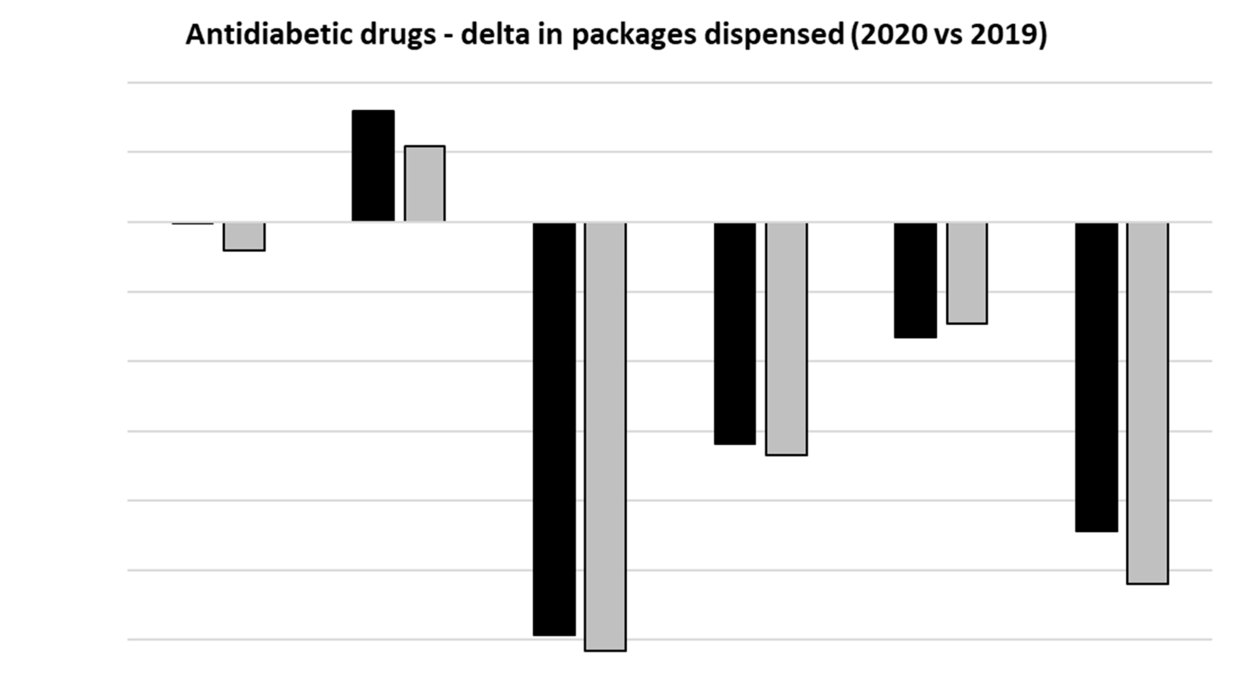

	ΔP1	ΔP2	ΔP3	ΔP4	ΔP5	ΔP6
■ Male	-0.05%	3.19%	-11.85%	-6.37%	-3.30%	-8.86%
□ Female	-0.80%	2.18%	-12.32%	-6.68%	-2.94%	-10.39%

(C) Antihypertensive drugs

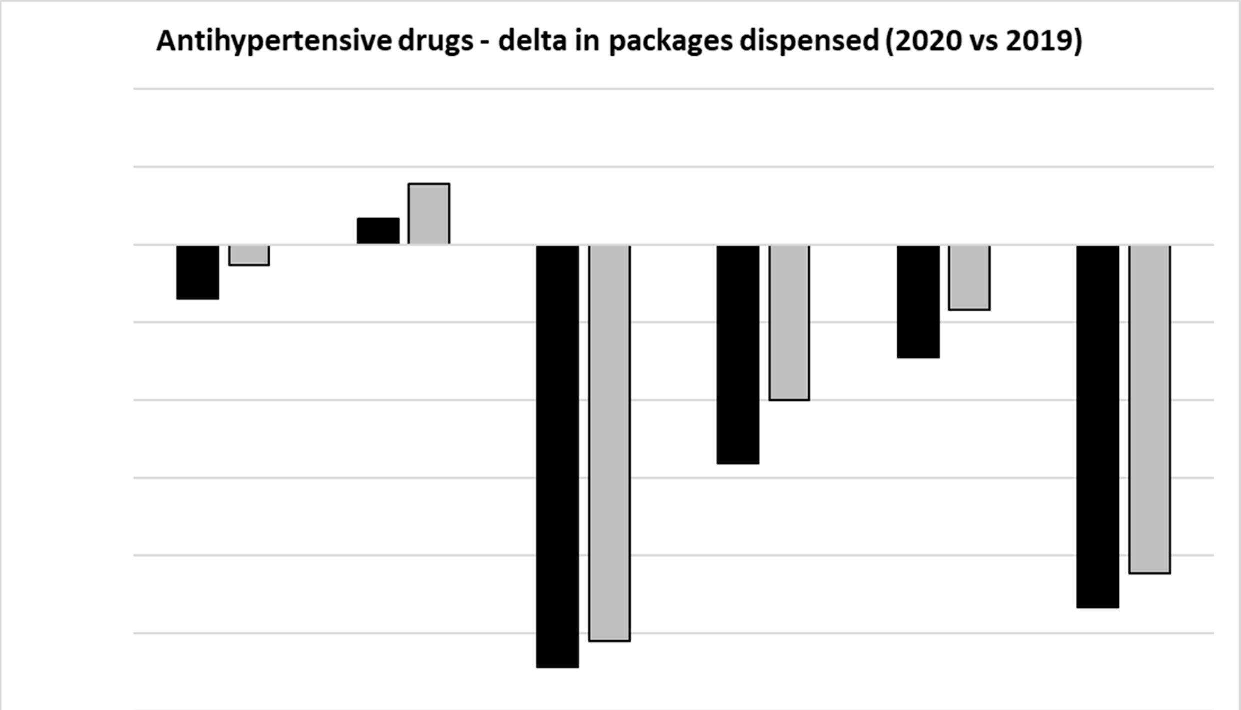

	ΔP1	ΔP2	ΔP3	ΔP4	ΔP5	ΔP6
Male	-1.39%	0.64%	-10.86%	-5.64%	-2.89%	-9.33%
Female	-0.53%	1.57%	-10.21%	-4.01%	-1.70%	-8.48%

Figure S4. Difference in dispensed packages per 1000 patients between study and control periods by age groups.

(A) Lipid-lowering drugs

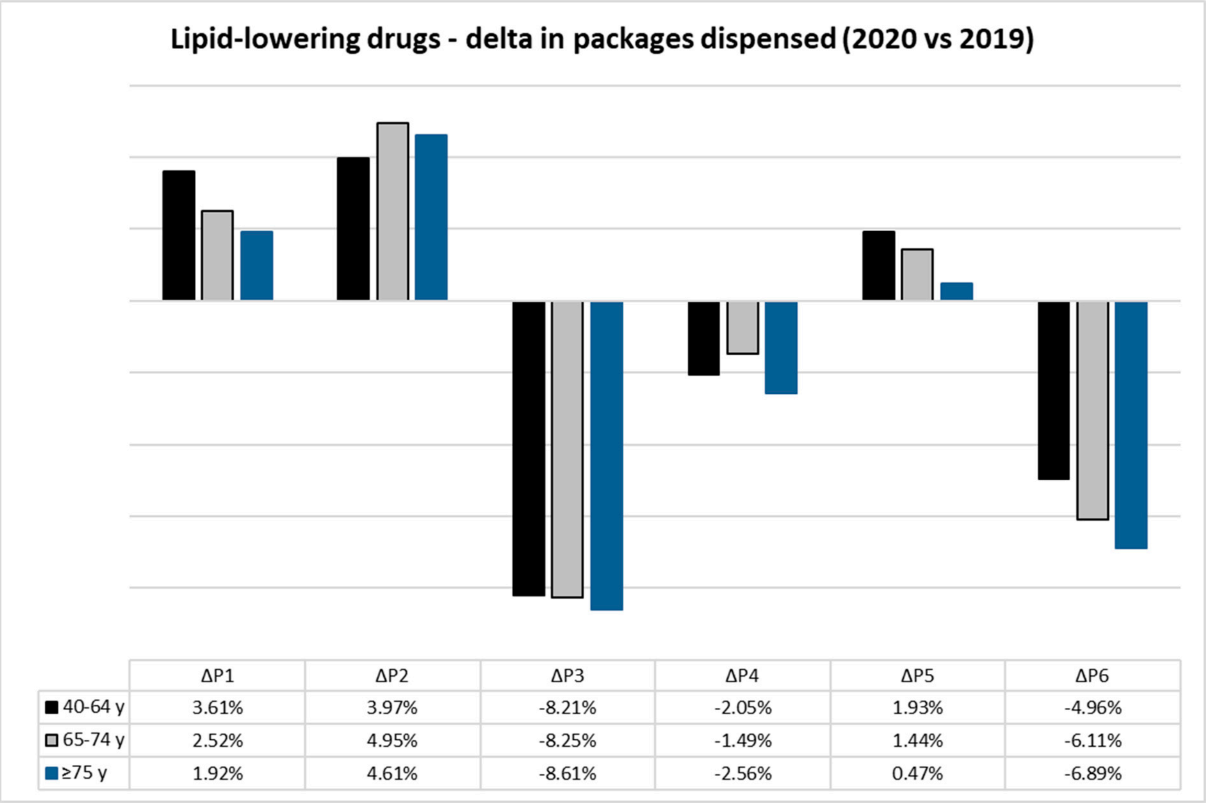

	ΔP1	ΔP2	ΔP3	ΔP4	ΔP5	ΔP6
■ 40-64 y	3.61%	3.97%	-8.21%	-2.05%	1.93%	-4.96%
□ 65-74 y	2.52%	4.95%	-8.25%	-1.49%	1.44%	-6.11%
■ ≥75 y	1.92%	4.61%	-8.61%	-2.56%	0.47%	-6.89%

(B) Antidiabetic drugs

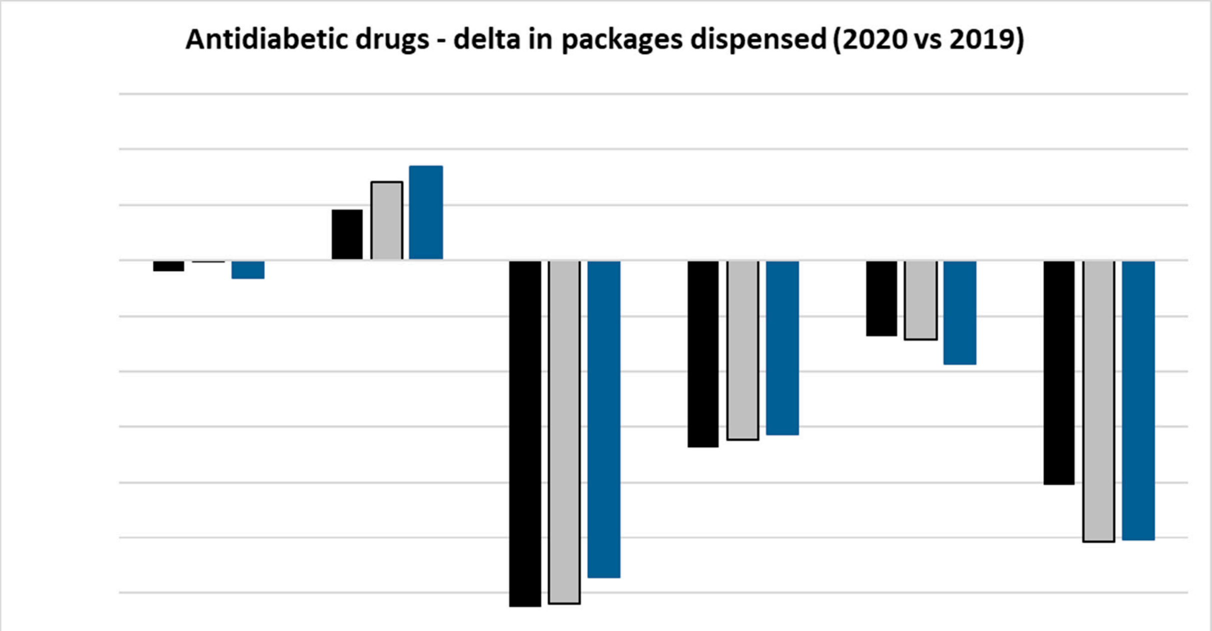

	ΔP1	ΔP2	ΔP3	ΔP4	ΔP5	ΔP6
■ 40-64 y	-0.38%	1.82%	-12.49%	-6.76%	-2.75%	-8.08%
□ 65-74 y	-0.02%	2.83%	-12.39%	-6.47%	-2.85%	-10.16%
■ ≥75 y	-0.63%	3.40%	-11.43%	-6.27%	-3.73%	-10.09%

(C) Antihypertensive drugs

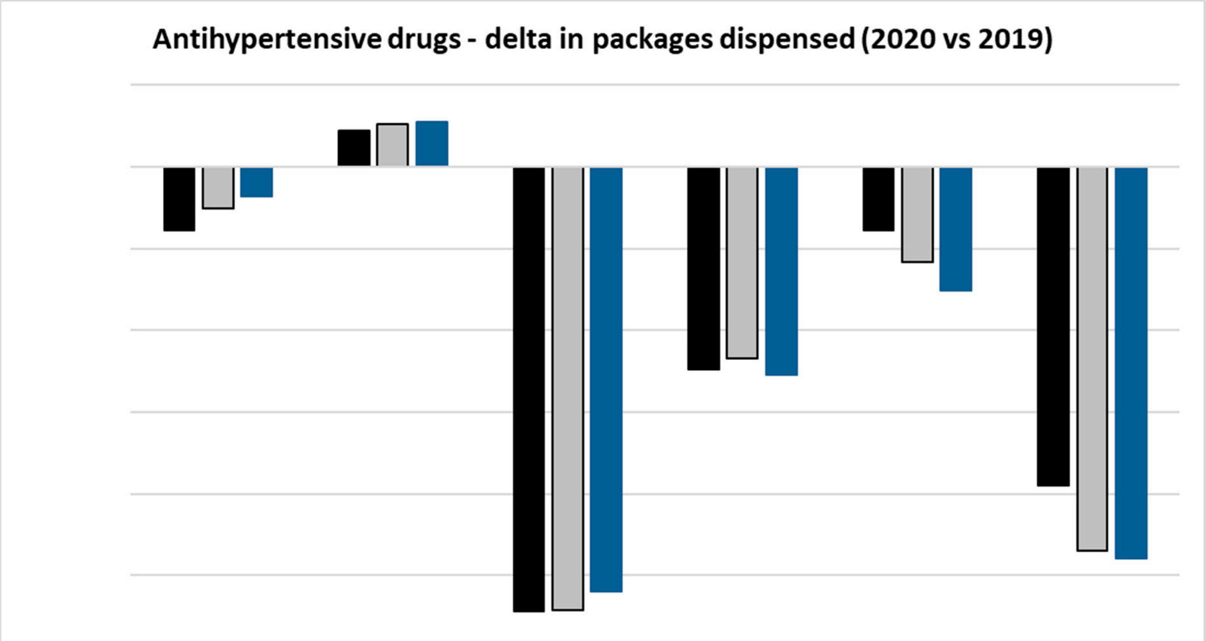

	ΔP1	ΔP2	ΔP3	ΔP4	ΔP5	ΔP6
■ 40-64 y	-1.54%	0.88%	-10.87%	-4.96%	-1.56%	-7.80%
□ 65-74 y	-1.01%	1.03%	-10.85%	-4.68%	-2.32%	-9.39%
■ ≥75 y	-0.72%	1.10%	-10.39%	-5.09%	-3.02%	-9.59%

Figure S5. Fifty two-week trends in chronic therapies dispensing in the population by metropolitan areas (those in dark blue represent areas with more COVID-19 cases recorded in 2020, while in light blue those with fewer cases)

(A) Lipid-lowering drugs

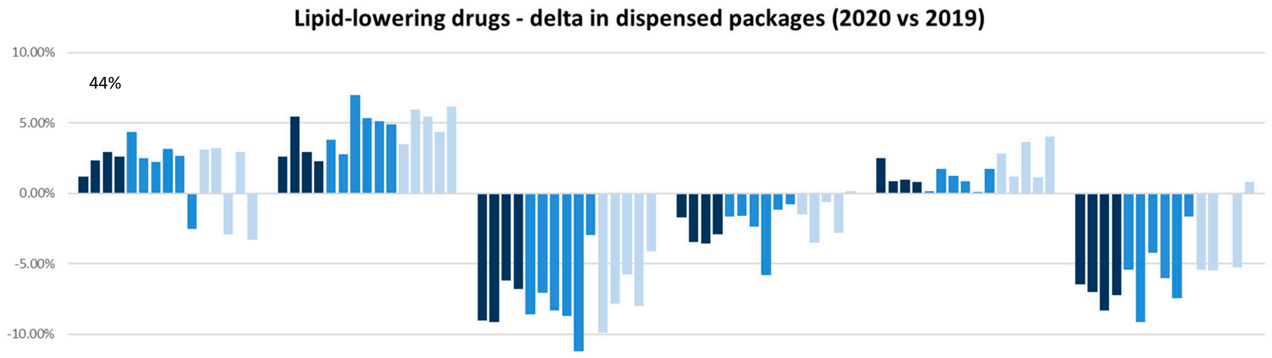

	ΔP1	ΔP2	ΔP3	ΔP4	ΔP5	ΔP6
	2.52%	2.81%	-7.03%	-3.04%	1.09%	-7.49%
	2.39%	4.86%	-9.68%	-1.52%	0.50%	-6.66%
	1.22%	5.86%	-7.62%	-1.85%	2.35%	-3.79%

(B) Antidiabetic drugs

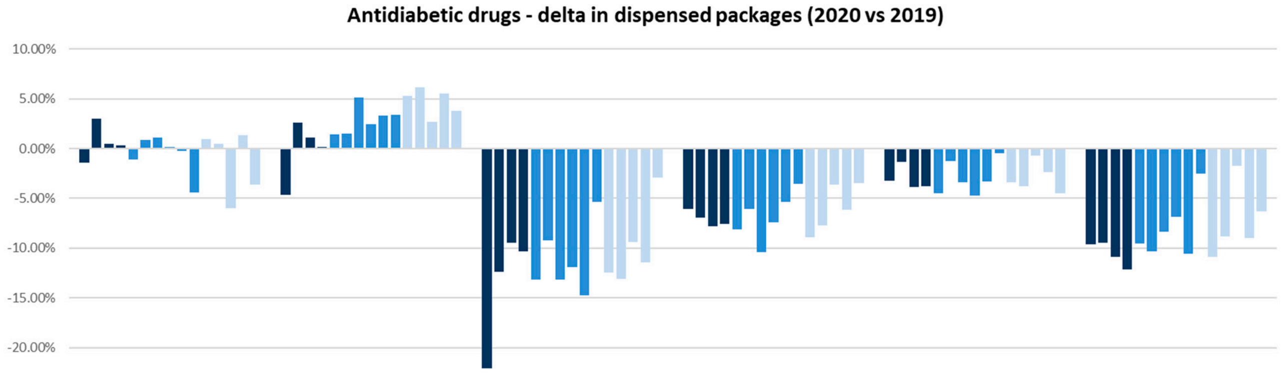

	ΔP1	ΔP2	ΔP3	ΔP4	ΔP5	ΔP6
	0.32%	0.06%	-11.74%	-7.41%	-3.56%	-11.18%
	-0.60%	3.02%	-12.98%	-5.96%	-3.01%	-10.97%
	-0.60%	4.95%	-10.81%	-6.48%	-2.94%	-8.22%

(C) Antihypertensive drugs

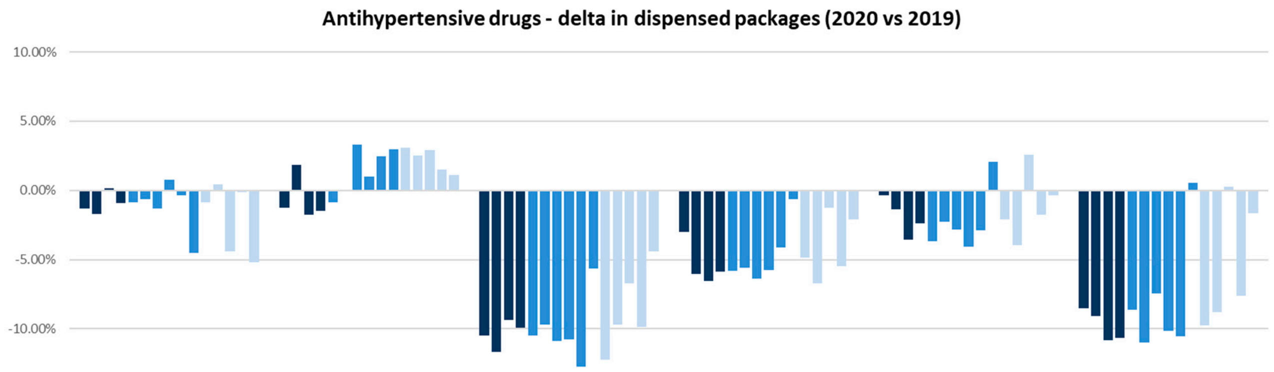

	ΔP1	ΔP2	ΔP3	ΔP4	ΔP5	ΔP6
	-0.65%	-1.30%	-9.95%	-5.73%	-2.45%	-10.31%
	-0.83%	1.87%	-11.45%	-4.39%	-2.59%	-9.25%
	-1.46%	2.28%	-9.54%	-4.57%	-1.57%	-6.84%
